# The Role of Magnesium in the Management of Cerebral Vasospasm

**DOI:** 10.1155/2013/943914

**Published:** 2013-05-21

**Authors:** Mitchell J. Odom, Scott L. Zuckerman, J Mocco

**Affiliations:** ^1^Vanderbilt University School of Medicine, Nashville, TN 37232, USA; ^2^Department of Neurological Surgery, Vanderbilt University School of Medicine, 1211 Medical Center Drive, Nashville, TN 37232, USA

## Abstract

Subarachnoid hemorrhage (SAH) is characterized by bleeding into the subarachnoid space, often caused by ruptured aneurysm. Aneurysmal rupture occurs in 700,000 individuals per year worldwide, with 40,000 cases taking place in the United States. Beyond the high mortality associated with SAH alone, morbidity and mortality are further increased with the occurrence of cerebral vasospasm, a pathologic constriction of blood vessels that can lead to delayed ischemic neurologic deficits (DIND). Treatment of cerebral vasospasm is a source of contention. One extensively studied therapy is Magnesium (Mg) as both a competitive antagonist of calcium at the *N*-methyl D-aspartate (NMDA) receptor, and a noncompetitive antagonist of both IP_3_ and voltage-gated calcium channels, leading to smooth muscle relaxation. In our literature review, several animal and human studies are summarized in addition to two Phase III trials assessing the use of intravenous Mg in the treatment of SAH (IMASH and MASH-2). Though many studies have shown promise for the use of Mg in SAH, there has been inconsistency in study design and outcomes. Furthermore, the results of the recently completed clinical trials have shown no significant benefit from using intravenous Mg as adjuvant therapy in the treatment of cerebral vasospasm.

## 1. Introduction

Subarachnoid hemorrhage (SAH) due to ruptured aneurysm occurs in 700,000 individuals a year [1], with nearly 40,000 of those cases occurring in the United States [2]. The mortality rate associated with the occurrence of SAH appears to have improved over the last 50 years [3], though it still remains that nearly half of all patients with a SAH will die within one month of the initial bleed [1]. Treatment of SAH is challenged by a number of complications. Cerebral vasospasm can occur in a majority of patients [4, 5] and is associated with poor outcome [5–8]. 

Despite having been recorded in patients treated for aneurysmal subarachnoid hemorrhage in 1951 [9], cerebral vasospasm (CVS) still remains a prevalent and morbid complication of SAH [10]. The occurrence of secondary ischemia and delayed ischemic neurologic deficits (DIND) is common sequelae in vasospasm and often leads to poor long-term outcomes. The diagnosis and treatment of CVS are made complicated by the heterogeneity of presentation and ambiguous etiology. Cerebral vasospasm is defined both clinically and angiographically, though these definitions are not mutually inclusive [11]. The occurrence of vasospasm is biphasic [1] with an acute phase that has been reported to begin 10 minutes [12] to 3-4 hours [13] after the initial bleed. The delayed or chronic phase begins 3–14 days after the initial bleed [13]. 

The cause of vasospasm, whether angiographic or clinical, is still a source of debate. The inflammatory response to SAH does appear to contribute to the etiology of cerebral vasospasm, specifically the role of leukocytes in releasing oxyhemoglobin after lysing extravasated erythrocytes in the subarachnoid space [14, 15]. Platelet aggregation has also been indicated in the etiology of CVS [16], indicating a link between a patient's Fisher grade upon presentation and the incidence of CVS. 

Effective interventions providing protection from CVS as well as improved outcomes post-CVS have lagged behind the treatment of SAH. Among the current treatment modalities for cerebral vasospasm, much interest has been placed in the use of calcium antagonists to prevent the contraction of smooth muscle in the cerebral arteries. The divalent inorganic cation magnesium is one such calcium antagonist and has been the subject of a large body of research.

Magnesium is one of the most common cations in the body and is required for many physiologic processes. It has been shown to cross the blood brain barrier in humans and animals [17–19], though serum levels are not reliable measure of cerebrospinal fluid (CSF) magnesium levels [20]. Magnesium administration in humans has been shown to be well tolerated but can lead to hypotension, bradycardia, and subsequent poor outcomes [21] if serum concentrations rise too high. 

In terms of a therapeutic role for magnesium in vasospasm, its function as a physiologic calcium antagonist and thus a potent vasodilator has been of most interest, though some neuroprotective effects have also been observed [21–24]. Mechanistically, magnesium functions in multiple physiologic processes throughout the body. Magnesium antagonizes the function of calcium by acting on and competing for the *N*-methyl D-aspartate (NMDA) receptors, causing an inhibition of excitatory postsynaptic potentials [20, 25, 26]. Magnesium also directly affects the physiologic function of calcium by serving as an intracellular noncompetitive inhibitor of the IP_3_-gated calcium channel [20] and a noncompetitive antagonist of voltage-dependent calcium channels [22]. Furthermore, magnesium appears to inhibit the formation of free radicals after tissue injury [27, 28]. The protection afforded to the neurovascular unit or to neuronal functioning is most likely due to the attenuation of the glutamate surge in a postischemic setting [29]. This prevents a postischemia excitotoxic phase from occurring and preserves brain parenchyma from further harm. Due to its ubiquitous functionality in the body, magnesium has been used in obstetrics to treat preeclampsia and in cardiology to treat acute myocardial infarctions [20]. 

When considering CVS, research into the use of magnesium as a therapeutic tool has focused mainly on its role in prophylaxis and its ability to treat or ameliorate established vasospasm. Intuitively, its use is indicated by the serum profile of patients who present with SAH. Hypomagnesemia occurs in approximately 10% of hospitalized patients, though this proportion rises sharply when other electrolyte deficiencies are present [20] and has been linked to poor outcomes and secondary ischemia when occurring after SAH [30]. 

The goal of this review is to assess the current state of research regarding magnesium, specifically its role as both a calcium antagonist and neuroprotective agent when used to treat cerebral vasospasm. To date, research on this topic has focused mainly on two domains: (1) experimental animal models and (2) human clinical trials. These two domains will be the main focus of this review, as they provide the greatest wealth of data to direct future studies. 

## 2. Experimental Data 

Magnesium has been well documented to have an effect on cerebral vasculature both *in vitro* [56–59] and in animal models [31, 33, 34, 36, 39, 44, 60, 61]. These studies are fairly heterogeneous in their methods and outcome measurements, though a trend can be seen towards improved outcome in treatment groups. B. T. Altura and B. M. Altura showed that excised basilar arteries (BA) displayed a contractile response to the removal of extracellular magnesium, and contrapositively, a dilatory response upon exposure to a high extracellular magnesium concentration [56]. These results were confirmed by Yang et al., also showing that low magnesium levels precede higher intracellular levels of calcium [59]. Many of the current studies use the dilation of the basilar artery (BA), vertebral arteries (VA), and superior cerebellar artery (SCA) as indicators of efficacy [31, 33, 34]. Additionally, a study by Hýža et al. [60] observed that vasospasm induced by surgical manipulation (axial tension) in rats was attenuated by topical application of magnesium on the affected arteries; though this study does not involve the cerebral vasculature, surgical treatment of aneurysms can replicate this type of tension in the brain and therefore may respond similarly.

In their review of animal models of SAH and pharmacologic treatment, Zoerle et al. reported that translation from animal models to clinical practice has proven challenging, an effect possibly due to publication bias, methodological flaws, or variability among studies [62]. They further report that intravenous magnesium sulfate administration was the only treatment to produce no reduction in angiographic CVS, whereas all other pharmacologic treatment modalities produced improved results. Upon further analysis, Zoerle et al. only included one study (conducted by MacDonald et al. [36]), involving the use of magnesium as a treatment in primates. 

To identify the role of magnesium in animal models, we have summarized several studies in Table 1, demonstrating magnesium's dilatory effects on vertebral arteries after established SAH and observed CVS. Of the seven studies published, only one failed to observe the dilatory effects of magnesium on cerebral vasculature. One study exclusively assessed the neuroprotective effects of magnesium sulfate after SAH, reporting a reduction in the size of post-SAbH ischemic damage [24]. 

In summary, it appears that intracisternal injection, as well as topical treatment of MgSO_4_, is successful in dilating cerebral vasculature [31, 33–35, 39]. Intravenous injection of MgSO_4_ appears to be less efficacious as a treatment, data that are in agreement with previous reports of the nonlinear transfer of Mg^2+^ between serum and CSF [19, 20, 63]. Doses of 0.5 mL/kg of 15 mMol MgSO_4_ appear to produce a profound effect when injected directly into the CSF; these data are in agreement with a dose-finding study, in which Mori et al. found that in order to produce a significant effect on cerebral vasculature, CSF levels of Mg^2+^ needed to be greater than 3 mEq/L [64]. 

The role of magnesium as a potential neuroprotective agent is more difficult to elucidate as only one study attempted to specifically assess this function, while other studies either provided a cursory mention or no mention of this function. Using a rat model of SAH, van den Bergh et al. set out to address the potential protective effects of magnesium when given prior to induced SAH. In their treated subjects, it was observed that magnesium reduced the total ischemic damage [24], potentially by attenuating the duration of neuronal depolarizations. This finding is consistent with Lin et al.'s [29] data reporting that administration of magnesium decreases the excitotoxic glutamate overload following ischemia. 

## 3. Clinical Data

Studies assessing the role of magnesium therapy in SAH and subsequent CVS have been hindered by inconsistencies across various clinical practices and a lack of large, multicenter studies. In both cases, several studies have yielded conflicting results, calling into question the quality of current data. Multiple variables account for the heterogeneity of the current state of magnesium therapy literature, including a variety of methods involving: (a) aneurysm treatment, (b) vasospasm surveillance, (c) magnesium administration times, (d) magnesium doses, and (e) outcome measures. All of these variables complicate the integration of data. 

A 2008 Cochrane Review aimed to determine whether calcium antagonists, specifically magnesium, improved outcome in patients with aneurysmal SAH [65]. All included studies were randomized controlled trials comparing calcium antagonists and/or a second calcium antagonist versus control. The final results yielded 3,361 total patients over 16 studies, three studies evaluating magnesium sulphate in addition to nimodipine. For magnesium in addition to standard nimodipine treatment, the relative risk was 0.75 for a poor outcome and 0.66 for clinical signs of ischemia. The authors concluded that magnesium is a promising new agent; however, more information is needed to draw definitive conclusions [65].

Four retrospective analyses were identified that analyzed the treatment of CVS with magnesium sulfate, though important clinical outcome data were not collected or omitted in two studies. In the other two, no significant difference in neurologic outcome was found in the treatment versus placebo groups. Aneurysm treatment modality was found to not have an effect on incidence of CVS, a finding that has been supported by a 600 patient study conducted by Alaraj et al. [66]. Overall, the general findings throughout the retrospective studies were that of slight neuroprotection offered by magnesium therapy as well as a trend towards decreased incidence of CVS. These data should be interpreted cautiously, however, especially after taking into account the prospective randomized controlled trials (RCT). 

There were eleven prospective studies identified for analysis in this review, including nine RCTs. Pilot studies indicate a possible benefit from the use of magnesium to prevent or treat CVS, though their results are mainly inconclusive. One study by Schmid-Elsaesser et al. [46] did not administer magnesium as an adjunct to the established calcium channel antagonist, nimodipine, instead of comparing the outcomes when each was administered separately. Only one study by Muroi et al. [45] reported a majority of adverse side effects from the administration of magnesium. All others reported very few complications in intravenous infusion. Similarly to the retrospective studies, a general trend can be elucidated: there is a progression towards decreased incidence of CVS in the magnesium group, though this is not always statistically significant. Reports from the prospective studies do present conflicting information, and many call for a large, multicenter trial to produce definitive conclusions.

Two recently completed Phase III trials are included in this analysis: Intravenous magnesium Sulphate for Aneurysmal Subarachnoid Hemorrhage (IMASH), and magnesium for Aneurysmal Subarachnoid Haemorrhage (MASH-2). Both were randomized, double-blind, placebo-controlled trials involving a large number of patients across multiple institutions. Primary outcome in the IMASH study was defined as either severe deficit identified using the Rankin Scale or death three months after treatment. No secondary outcomes, quality of life measurements, or neurocognitive deficits were assessed. For the MASH-2 study, primary outcome was defined as good outcome using the Glasgow Outcome Scale Extended questionnaire and did secondary outcomes such as the presence of CVS and the SF-36 questionnaire score. Despite differences in outcome measures for these two clinical trials, similar outcomes were found for both magnesium and placebo groups in both studies. In the IMASH trial, Wong et al. concluded that intravenous magnesium treatment was not efficacious in the treatment of SAH [41]. In MASH-2, Mees et al. have not recommended the use of magnesium as an adjunct in SAH [40]. Despite significant strengths, both studies suffered from methodological flaws. Two important limitations were as follows: (1) recruitment of patients was completed over a wide range of times, and (2) initiation of treatment was consistently reported to begin after the acute CVS window had passed. In the IMASH and MASH-2 studies, participants were included if they were admitted within four days and 48 hours after SAH, respectively. This time frame excludes the possibility of utilizing magnesium as a prophylactic tool to prevent acute CVS. Furthermore, though the MASH-2 study did report serum levels of magnesium during treatment, this was not recorded in the IMASH study. This omission is important as the nonlinear movement of magnesium from the blood into the CSF might not have allowed a therapeutic amount of magnesium to accumulate and be maintained in the CSF. However, despite their deficits, these are the first studies to record a large number of patients and long-term outcomes without a loss of followup. Studies included in this review are summarized in Table 2.

## 4. Future Directions

While the value of intravenous magnesium therapy for the prevention and treatment of CVS remains questionable, magnesium may still play a role in the course and eventual treatment of this disease. Risk-benefit analyses of various alternative administration routes need to be considered, such as intracisternal or intrathecal infusion. These studies also may indicate a different role for CVS in mortality and morbidity following SAH. There may be other causes of DCI or DIND that are not directly related to vasospasm, and research into alternative etiologies, and how those alternative etiologies may confound investigations into magnesium, is warranted. 

## Figures and Tables

**Table 1:** Summary of *in vivo* animal models of SAH/CVS and magnesium therapy.

Author/date	Animal type	SAH model used	Mg^2+^ administration location	Mg^2+^ administration dose	N treatment/ placebo	CVS assessment modality	Major outcome
Mori et al., 2012 [31]	Canine	Double hemorrhage [32]	Intrathecal	0.5 mL/kg of 15 mMol MgSO_4_	7/NA	Angiography	BA, VA diameter significantly increased after injection
Mori et al., 2011 [33]	Canine	Double hemorrhage [32]	Intracisternal	0.5 mL/kg of 15 mMol MgSO_4_	7/NA	Angiography	BA, VA, and SCA diameter significantly increased. With adequate CSF Mg level
Mori et al., 2009 [34]	Canine	Double hemorrhage [32]	Intracisternal	0.5 mL/kg of 15 mMol MgSO_4_	10/NA	Angiography	BA, VA, and SCA diameter significantly increased
Mori et al., 2008 [35]	Rat	Double hemorrhage [32]	Intracisternal	1.5 *μ*L/min for 30 minutes of 10 mMol MgSO_4_	8/6	Autoradiographic measurement of cerebral blood flow	Mg^2+^ group showed improved cerebral blood flow in 12/16 locations
MacDonald et al., 2004 [36]	Primate	Single hemorrhage [37]	IV	−0.086 g/kg bolus −0.028 g/kg/day	5/5	Angiography	No angiographic evidence of MgSO_4_ effect on CVS
van den Bergh et al., 2002 [24]	Rat	Sheffield model [38]	IV	90 mg/kg MgSO_4_	14/19	N/A	Pretreatment with MgSO_4_ reduces the size of SAH-induced ischemic damage
Ram et al., 1991 [39]	Rat	Single hemorrhage [37]	Group 1: topical Group 2: IV	Group 1: 10 mEq/L Group 2: not stated	Group 1: 30 Group 2: 20 Placebo: 10	Computerized image analysis of basilar artery	Group 1: BA dilated to 150% of baseline Group 2: BA dilated to 75% of baseline

**Table 2:** Clinical data regarding the use of magnesium therapy in CVS.

Author/ date	Study type	Mg^2+^ administration type	N treatment/ placebo	Major results
Mees et al., 2012 [40]	RCT	64 mMol/day (IV) of MgSO_4_ for 20 days	606/597	Outcomes assessed by the modified Rankin Scale were similar in the treatment and placebo groups.

Wong et al., 2010 [41]	RCT	20 mMol bolus *followed by* 80 mMol/day (IV) for 14 days	169/158	(i) Outcome measured by GOS was the same at six months after treatment (ii) Incidence of CVS was similar in both groups

Westermaieret al., 2010 [42]	RCT	16 mMol bolus of MgSO_4_ *followed by* 8 mMol/hour continuous infusion for 10 days	55/55	(i) Lower incidence of CVS in Mg^2+^ group (ii) Lower incidence of delayed ischemic infarction in Mg^2+^ group (iii) DIND nonsignificantly reduced in Mg^2+^ group, though fewer patients with DIND expressed delayed ischemic infarction

Shah et al., 2009 [43]	Retrospective case series	8–32 mMol (IA) via super-select catheterization	14	(i) No long-term outcomes reported (ii) No cerebral infarction in 12/14 patients

Mori et al., 2009 [44]	Prospective case series	15 mMol/L MgSO_4_ at 20 mL/hour (intracisternal) for 20 days	10	(i) Five patients had good recovery (ii) One patient exhibited moderate disability (iii) One patient exhibited severe disability (iv) Two patients progressed to a vegetative state (v) One patient died

Muroi et al., 2008 [45]	RCT	16 mMol bolus in 150 mL *followed by *64 mMol continuous infusion for 12 days	31/27	(i) Magnesium treatment had to be discontinued in 52% of patients due to adverse side effects (ii) Trend towards improved outcome observed

Dorhout Mees et al., 2007 [21]	Retrospective case series	64 mMol/day for 20 days	155/194	(i) Risk of DCI was lower in patients with higher serum magnesium concentrations when compared to the lowest quartile (ii) No effect on incidence of poor outcome

Schmid-Elsaesser et al., 2006 [46]	RCT	10 mg/kg bolus *followed by *30 mg/kg (IV) infusion of MgSO_4_ for 7 days	53/51	(i) No difference in outcome measured by GOS after 12 months (ii) Similar incidence of CVS in both groups (iii) Rate of cerebral infarction similar in both groups.

Wong et al., (2006) [47]	RCT	20 mMol bolus *followed by* 80 mMol/day (IV) for 14 days	30/30	(i) CVS incidence decreased, but not statistically significant (ii) Vasospasm detected via TCD shorter in duration (iii) No difference in outcome measured by GOS at 6 months

Prevedello et al., 2006 [48]	RCT	20 mMol bolus of MgSO_4_ *followed by* 120–150 mMol/day	48 treated with nimodipine, Triple-H therapy, bed rest/ 24 treated with MgSO_4_ adjunct	(i) Incidence of vasospasm was reported to be equal in both groups. (ii) Vasospasm occurring in the nimodipine-only group was correlated with longer hospital stays, when compared to the MgSO_4_ adjunct group

Stippler et al., 2006 [49]	Retrospective historically controlled.	100 mMol MgSO_4_/day continuous infusion	38/38	(i) Incidence of vasospasm in the Mg^2+^ adjunct group decreased by 18% (ii) Outcome not changed in Mg^2+^ group (*P* > 0.05)

Yahia et al., (2005) [50]	Prospective pilot study	100 mMol/hour MgSO_4_ for 10 days	19	(i) No adverse effects from continuous magnesium infusion (ii) Lower incidence of both angiographic and clinical CVS observed than the literature values

van den Bergh, 2005 [51]	RCT	64 mMol (IV) MgSO_4_ for 14 days	139/144	Suggested benefit for reduction of DCI, though results were inconclusive

Venya et al., 2002 [52]	RCT	6 g bolus *followed by* 2 g/hour MgSO_4_ for 10 days	20/20	(i) No significant reduction in incidence of CVS (ii) Trend toward improved neurological outcome measured by GOS at 3 months

Chia et al., 2002 [53]	Retrospective case series	24–52 mMol/day continuous infusion MgSO_4_	13/10	(i) Significant reduction in the incidence of CVS (ii) Neurologic outcome was similar in both groups

Wong et al., 2011 [54]	Meta-analysis	—	441	(i) Lowered odds ratio for incidence of CVS and DCI in the magnesium treatment groups (ii) Increased odds ratio for favorable outcomes

Wong et al., 2010 [41]	Meta-analysis	—	875	(i) No benefit from magnesium infusion on the incidence of cerebral infarction (ii) Nonsignificant increase for odds ratio of favorable outcome at 3 and 6 months.

Chen and Carter, 2011 [55]	Meta-analysis	—	936	(i) Decreased risk of poor outcome at 3–6 months in the magnesium treatment groups (ii) Risk of mortality after SAH was unaffected

Ma et al., 2010 [22]	Meta-analysis	—	699	(i) Magnesium infusion reduced the risk for DCI and poor outcome after SAH (ii) Serum levels need to be monitored closely to prevent adverse side effects
